# Origin of electrochemical voltage range and voltage profile of insertion electrodes

**DOI:** 10.1038/s41598-024-65230-x

**Published:** 2024-06-21

**Authors:** Elham Shahpouri, Mohammad Mahdi Kalantarian

**Affiliations:** Department of Ceramic, Materials and Energy Research Center, PO Box 31787-316, Karaj, Iran

**Keywords:** Operating potential range, Voltage window, Intercalation batteries’ electrode, Underlying mechanism, Voltage profile, Batteries, Batteries

## Abstract

This study evaluates electrochemical voltage-range and voltage-profile regarding electrodes of insertion (intercalation) batteries. The phrase “voltage-range” expresses the difference between obtained maximum and minimum potential for the cells. It also can be called as operating voltage-range, working voltage-range, electrochemical voltage-range, or voltage window. This paper proposes a new notion regarding electron density of states, i.e. trans-band, which can be implemented to justify the voltage -range and -profile, by means of Fermi levels’ alignment. Voltage -range and -profile of a number of insertion electrode materials are clarified by the proposed theoretical approach, namely LiMn_2_O_4_, Li_2_Mn_2_O_4_, ZnMn_2_O_4_, LiFePO_4_, LiCoO_2_, Li_2_FeSiO_4_, LiFeSO_4_F, and TiS_2_. Moreover, the probable observed difference between charge and discharge profile is explained by the approach. The theoretical model/approach represents a number of important concepts, which can meet some scientific fields, e.g. electrochemistry, energy storage devices, solid state physics (DFT), and phase diagrams. By means of DFT calculations, this paper deals with quantizing the energy of electrochemical reactions, justifying the configuration of voltage-profile, and explaining the origin of the voltage-range. Accordance with the experimental observations suggests that this paper can extend boundary of quantum mechanics toward territories of classical thermodynamics, and boundary of the modern thermodynamics toward kinetics. Opening a new horizon in the related fields, this paper can help tuning, engineering, and predicting cell-voltage behavior.

## Introduction

Voltage -range and -profile are the critical properties to control the performance and durability of energy storage systems. Nowadays, the importance of energy storage devices and its impact on human’s life is well-known and the growth of their demand is accelerated. Development of a number of high demanded new technologies depends on improvement of energy storage devices, i.e. batteries^1^. Each required equipment needs its own operating voltage-range. For instance, one of the most important new and vital technologies is electric (EV) or hybrid-electric (HEV) vehicles^2,3^. To be sure about almost constant operation of EVs and HEVs, a high voltage and narrow voltage-range can be useful for such an application. To inform, the current used electrode materials in their cells usually are LiNi_1–x–y_Mn_x_Co_y_O_2_ (NMC)^4^, LiNi_1–x–y_Co_x_Al_y_O_2_ (NCA)^5^, and LiFePO_4_ (LFP)^6,7^, which their voltage-ranges are 3.7–4.3^4^, 3.6–4.2^5^, and 3.3–3.6 V^6,7^, respectively. Also, a high voltage and narrow voltage-range might be important for storage of renewable energies (other important technological demands). On the other hand, a suitable wide range of voltage can be more effective to detect the state of charge (SOC)^8^. Appropriate detecting SOC is more important in some demanded technologies, such as portable electronic devices. Accordingly, tuning the operative voltage-range as well as understanding its underlying mechanisms are vital and considerable^9^.

One of the most promising energy storage devices, especially for high scale applications, is lithium-ion batteries (LIBs). Therefore, electrode materials of LIBs are considered as important case studies in this paper. The first commercially used LIB cathode, LiCoO_2_ (LCO), presented electrochemical voltage-range of ~ 3.9–4.3 V^10^. Afterwards, introducing LFP with a narrow voltage-range (3.3–3.6 V)^6,7^ has attracted a great attention from the researchers all over the world. It seems that narrower voltage-range can be more favorable, since it ensures accomplishing the reaction before reaching cut-off voltage even in higher rates, increasing battery efficiency^11,12^. Also, considering the operating voltage of the electronic components, the narrow voltage-range simplifies designing and enhances durability of electronic circuits. Subsequently, some researches have investigated to understand the origin and underlying mechanisms for the narrow voltage-range of LFP, e.g. domino-cascade mechanism^13,14^, traditional shrinking core model^15–17^, anisotropic shrinking core model^18^, spinodal decomposition^19^, etc. Considering the cell-voltage related to difference between energy of lithiated and delithiated phases, LFP has been known as a binary phase electrode^20^. The attempts to find mechanisms of the narrow voltage-range of LFP declare the importance of the subject. Also, it shows that wider range of voltage is more reasonable in the researchers’ opinion. It can be due to effect of existence of solid solution (concentration gradient) in the electrochemical reaction between the phases. Moreover, historically, the previous batteries exhibited wider voltage-ranges.

Existence of the concentration gradient between the reacted phases is based on diffusion process concept. In our previous work^21^, we considered the concentration gradient to justify observed voltage behaviors of the electrode materials. We have proposed that there should be main reaction phases and their concentration gradient region would be formed between the main phases or in the interface, originating voltage deviation from its ideal amount. Accordingly, a number of important phenomena and observations cannot be clarified without considering this concept, namely non-ideal voltage behavior^21^, bipolarization mechanism^11^, voltage hysteresis^11^, first cycle over voltage^11^, memory effect^22^, boundaries of charge/discharge curvatures^12^, voltage overshooting^23^, etc. Nevertheless, in the mentioned models, origin of the voltage-range and the corresponding main phases have not been discussed.

Very recently, it has been proposed that the voltage of an electrochemical cell can be estimated by difference between Fermi energies of the reactants and products^24,25^. This concept was based on another suggestion which discussed rate-capability of battery electrode materials^26^, considering reacted and unreacted phases as semiconductors and their joint as a semiconductor junction^26^. In any case, the most important concept is Fermi levels’ alignment of the intercalated and deintercalated structures. In fact, the alignment of Fermi levels is the quantum mechanical equivalent of the classical conservation of energy. As a matter of fact, this paper unifies our previous two branches of investigation fields (based on modeling^21,27–29^ and theoretical DFT approaches^26,30,31^).

In this work, we establish a methodology to find the origin of voltage-range. We used the phrase of “*voltage-range*” to express the difference between obtained maximum and minimum potential (voltage) of the electrodes. It can be called as operating voltage-range, working voltage-range, electrochemical voltage-range, or voltage window. On the journey of finding voltage-range, we reach an approach to predict voltage-profile of different electrode materials and its configuration. As a matter of fact, the electrode potentials are equal to the work done in transferring electrons and ions from infinity to the reaction sites, i.e. the interior of the solid phases. After all, importance of this work is explaining the classical physics’ concept of electrochemical voltage with means of modern physics, i.e. quantum mechanics. Considering the cell-voltage related to energy difference between the reaction phases, this paper can be a clue to find origin of phase diagrams.

## Methodology

### Computational details

All the calculations in this study were performed using the full-potential linear augmented plane-wave (FP-LAPW) method as implemented in the WIEN2K^32^ code within the framework of density functional theory (DFT)^33^. The convergence of the self-consistent iterations was performed within 0.0001 Ry. Before running the self-consistency cycle (SCF) calculations, the experimentally obtained initial structures were fully relaxed. The relaxation process was carried out, including (i) geometry minimization of the atomic positions according to the inter-atomic forces, (ii) optimization of the volume with a constant a:b:c ratio, and (iii) optimization of the ratios between the cell parameters (if it was operational). The initial structures for LiMn_2_O_4_^34^, Li_2_Mn_2_O_4_^35^, ZnMn_2_O_4_^34^, LiFePO_4_^26^, LiCoO_2_^36^, Li_2_FeSiO_4_^37^, LiFeSO_4_F^38^, and TiS_2_^39^ were taken from the corresponding cited references and the space groups were $\text{Fd}\overline{3}m$, $I{4}_{1}/amd$, $\text{Fd}\overline{3}m$, $Pnma$, $R\overline{3}m$, $Pmn{2}_{1}$, $C2/c$, and $P\overline{3}m1$, respectively. For these structures, integrals were calculated over the Brillouin zone with k-points based on $7 \times 7 \times 7$, $7 \times 7 \times 7$, $5 \times 8 \times 11$, $14 \times 14 \times 2$, $6 \times 8 \times 8$, $7 \times 4 \times 7$, and $9 \times 9 \times 4$ Monkhorst–Pack (MP) for the above-mentioned materials, respectively. The calculations were carried out using spin-polarized Perdew-Burke-Ernzerh generalized gradient approximation (PBE-GGA) plus an on-site Coulomb self-interaction correction potential, hereinafter called as GGA + U^40^. For more comprehensive assessments, the Hartree–Fock method of Exact-exchange as Hybrid functional^41^ (HF) and local spin density approximation (LSDA) were also used for a number of structures.

The major spin was set as -up. The U value for calculation of LiMn_2_O_4_ cathode material with spinel structure was considered to be 3 eV for GGA + U and LSDA + U. The U values used for ZnMn_2_O_4_, LiFePO_4_, LiCoO_2_, Li_2_FeSiO_4_, LiFeSo_4_F, and TiS_2_ electrode materials were 0.563, 6, 5.5, 4.5, 5, and 5.5 eV, respectively.

To expand the wave functions in the interstitial region a plane-wave cut-off value of K_max_.R_mt_ = 7.0 was used, where R_mt_ is the smallest atomic sphere radius in the unit cell and K_max_ is the magnitude of the largest K vector. The Fourier-expanded charge density was cut at ${G}_{max}= {12 (Ryd)}^{1/2}$. The maximum value of the angular momentum (l_max_) was set to 10 for the wave function expansion inside the atomic spheres. Additional details are given in the Supporting Information (S.I).

### Criteria and assumptions

There are a number of basic rules and assumptions that should be recalled while considering a calculated density of states (DOS) diagram in view of the herein proposed approach, including:

1. Spin is an inherent property of an electron. Therefore, an electron with spin -up (down) cannot transfer to a spin -down (up) band in a DOS diagram of the same structure^31^ and also of a connected (joined) structure.
2. Joined intercalated-deintercalated (reacted-unreacted) structures should be considered as semiconductor junctions^26,30,31,42^.
3. The reacted-unreacted junction can be considered one-dimensional in the interface of the junction^21,26,43,44^.
4. In the electrically joined/connected semiconductors, the Fermi level should be aligned in the entire body of the joined materials in their equilibrium state. Accordingly, for our evaluations, the Fermi level should be aligned in the DOS diagrams of the reacted and unreacted structures.
5. The net component of Li^+^ ion motion can be considered parallel to the electric field direction. This means that thermal disorder can be deliberated as a 2nd order perturbation.
6. The applied current rate in lithiation/delithiation process is not limited by neither Li^+^ ion mobility, nor electron conductivity. In other words, ion- and electron- conduction can endure the applied current rate.

## Results and discussion

The here proposed theoretical approach is based on a number of physics’ laws and some of their special infer which can be applied in DOS diagrams for the intercalation electrodes in their intercalated and deintercalated states. Noteworthy, we consider Li-ion electrode materials as very important case studies; however, due to generality of our basis and utilized laws, the approach can be generalized for other insertion cells and even other analogous electrochemical systems.

Noteworthy, the paper aimed to introduce theoretical concepts to find origin of electrochemical behaviors of electrode materials. Therefore, the approximation methods of the calculations, or even the entire DFT, are just the evaluation tools of the paper to establish its concepts.

We chose the best approximation method for each material to calculate DOS diagrams in the DFT calculation framework. As a matter of fact, in DFT calculations the best approximation should be the one that consents with the experimental values. It was assumed that the here chosen calculated DOS diagram was relevant for each considered material. For instance, here, we concluded that the results of GGA + U = 6 eV approximation were appropriate for the LFP case study. Nevertheless, if an approximation was not relevant for a case study, then a relevant DOS should be used instead.

As an additional point, dealing with semiconductor materials, the precise stating of so-called *Fermi levels* is factually *Fermi energies*; since, there are no levels inside band gaps. However, in some parts of the paper, we still use *levels* instead of *energies* to imaginate the alignment/movement process.

For better comprehending the approach, let’s explain the conceptual model with some case studies. A number of important Li-ion electrode materials are evaluated in this paper, namely LiMn_2_O_4_ (LMO), LiFePO_4_ (LFP), LiCoO_2_ (LCO), Li_2_FeSiO_4_ (LFS), LiFeSO_4_F (LFSF), ZnMn_2_O_4_, and LiTiS_2_. Some important information is discussed in the main text, while the other related data are given in S.I.

### Introducing trans-band and operational trans-band

Equation (1) shows de/lithiation (oxidation/reduction) reaction of a well-known electrode material, i.e. LMO.

1 $${\text{LiMn}}_{2} {\text{O}}_{4} \leftrightarrow {\text{Mn}}_{2} {\text{O}}_{4} + {\text{Li}}^{ + } + {\text{e}}^{ - }$$

**Figure 1:**
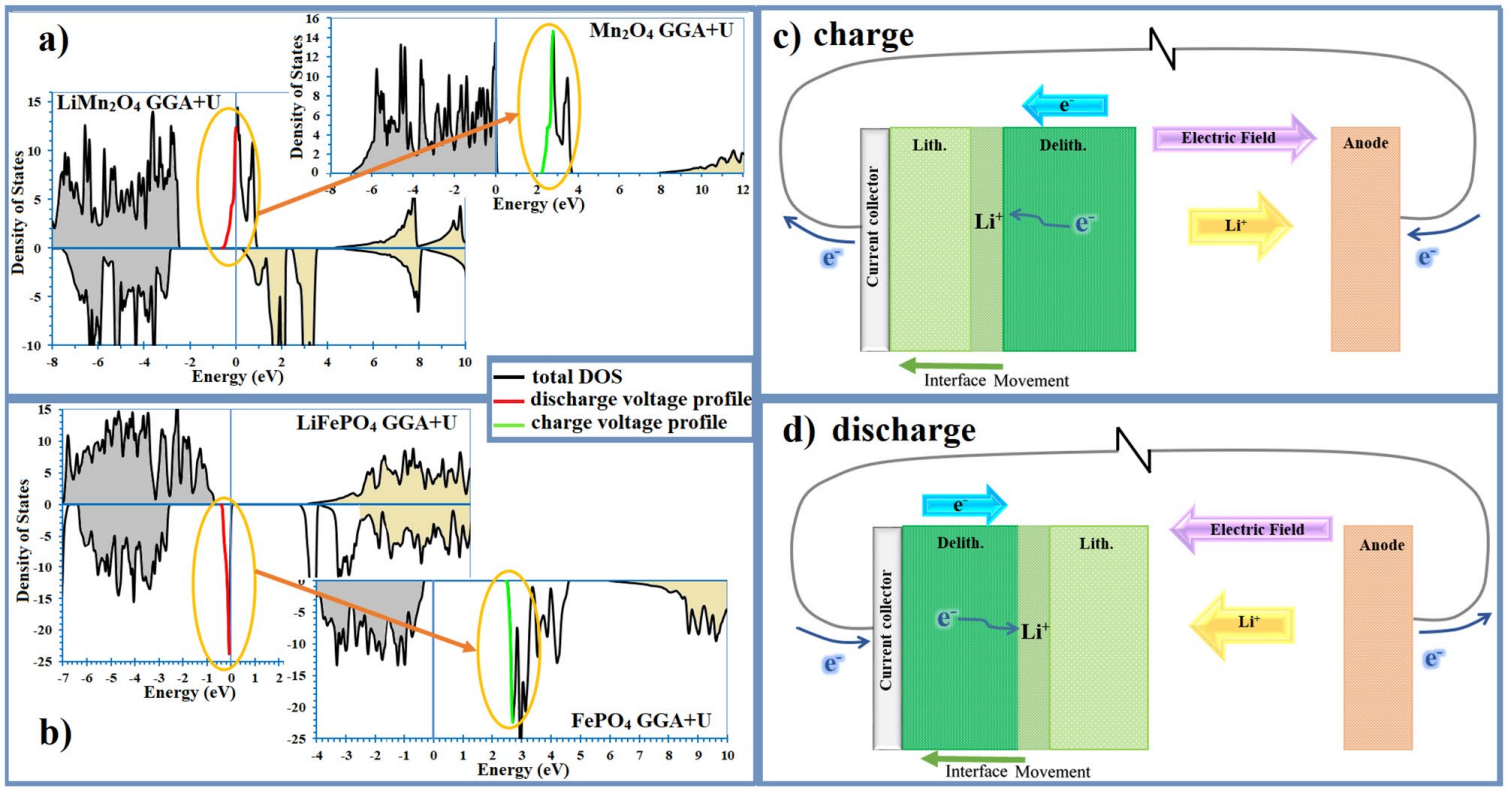
DOS diagrams of (**a**) LMO and (**b**) LFP cathode material in the lithiated and delithiated states, to show operational trans-bands. The trans-bands of lithiated and delithiated structures are shown by red and green colors, respectively, and their shift from beneath to above the Fermi level is illustrated by the orange arrow. Also, schematic of the (**c**) charging and (**d**) discharging process of a cell are given, illustrating the configuration of the delithiated (Delith.) and lithiated (Lith.) structures, showing electron transfer through the delithiated into lithiated structure within the interface.

In the charging reaction (left to right, delithiation, oxidation), valance state of the transition metal, Mn, changes from + 3.5 to + 4. Accordingly, an occupied orbital of half of the Mn atoms should be unoccupied under the charge reaction. Consequently, it is predictable that a band in DOS diagram transfers from occupied (beneath Fermi level) to unoccupied band (empty, above Fermi level) during delithiation process. Such a band can be seen in Fig. 1a. In the figure, the DOS bands of the lithiated state, related to *3d* orbitals, are crossed by the Fermi level and moved totally above the Fermi level for delithiated state (Fig. 1a). It is the closest band to the Fermi energy before and after delithiation. We name this band as *“trans-band”*, showed in Fig. 1 for LMO. Actually, the trans-band is the maximum valance state of the lithiated structure; whereas, it is the closest conduction band of the delithiated structure, i.e. the conducting electrons of the delithiated structure should firstly fill this band. We proposed that the conducting electrons in the trans-band of the delithiated structure should be moved to the trans-band of the lithiated structure during the lithiation (discharge) process. In other words, the lithiation process coincides with the conversion of delithiated trans-band to lithiated one. The inverse process should be taken for the delithiation (charge) process.

Conducting electrons should transfer through the delithiated structure to make conduction and perform the reaction in the delithiated-lithiated junction^25^ (see Fig. 1c,d).

In contrast with LMO, in some cases, the whole trans-band did not participate in the de/lithiation process. We name the active part of trans-band as “*operational trans-band*”. For instance, Fig. 1b illustrates DOS diagram of LFP. As it can be seen, the entire peak of trans-band of the lithiated state is located under the Fermi level. In this case, the lower energy of the trans-band outline (the left side of the trans-band in Fig. 1b) should be considered as the operational trans-band. To explain rational of the operational trans-band, two conditions should be satisfied to determine the active part of the trans-band. It should be i) corresponded to the number of reacting electrons, and ii) energetically favorable (having the lowest energy).

Since calculated DOS diagrams are related to one unit-cell and due to quantum mechanics concepts (uncertainty principle), reacting electrons should be taken into account as Eq. (2).

2 $${OTB}_{DOS}={\Delta n}_{VS}^{e}\times {n}_{d}^{e}\times {n}_{uc}^{TM}$$

where, ${OTB}_{DOS}$ is the DOS value of operational trans-band, ${\Delta n}_{VS}^{e}$ is the difference of valance states of transition metal (TM) within lithiated and delithiated structures, ${n}_{d}^{e}$ is the number of electrons within *d* orbital, and ${n}_{uc}^{TM}$ is the number of TM atoms within the unit-cell.

For more explanations, the operational trans-band corresponds to the reacting electrons in the considered electrochemical reaction, however, within the DOS diagrams. For instance, in Eq. (1), an electron participates in the reaction formula; while, other parameters should be considered to determine the state of this electron in the trans-band. In this way, the reacting electron supplies from two Mn (transition metal) atoms per formula (LiMn_2_O_4_), leading to ${\Delta n}_{VS}^{e}$ in Eq. (2). Obviously, the Mn atoms can supply the reacting electron from their valance orbitals (*3d* orbitals), i.e. 5 electrons for Mn. The quantum mechanics’ concepts (based on uncertainty principle) suggest that all the valence electrons have probability of contributing in the reaction, leading ${n}_{d}^{e}$ in Eq. (2). Furthermore, in the DFT calculations DOS diagrams are calculated for one unit-cell. Therefore, the number of transition metals in the unit-cell (${n}_{uc}^{TM}$) should be also taken into account. These three parameters are the origin of Eq. (2).

For examples, operational trans-band of LMO and LFP should be obtained by Eqs. (3) and (4), respectively. Accordingly, DOS values of operational trans-band for LMO and LFP would be 10 and 24, respectively. Interestingly, these amounts are almost equal to the left outlines of the trans-band peaks, which also satisfies the lowest-energy, i.e. condition ii.

3 $${OTB}_{DOS}^{LMO}=\left(4-3.5\right)\times 5\times 4=10$$

4 $${OTB}_{DOS}^{LFP}=\left(3-2\right)\times 6\times 4=24$$

### Justifying voltage-range and voltage-profile

To move forward into the concept, it should be kept in mind that energy and density of states values in the DOS diagrams correlate with voltage and capacity, respectively. Previously, we have established that difference between the energy levels in eV unit is correlated with cell-voltage in Volt unit^24^. It has been deliberated for both of the voltage approximation approaches, i.e. Fermi and internal energy^24,45,46^. Regarding the relation of DOS values and capacity, correspondence of the reacting electrons with the reacting component (e.g. Li) imposes direct correlation between DOS and capacity.

In the reacted and unreacted structures of the electrochemical cells, Fermi levels of the structures should be aligned^24,47^. As a matter of fact, we are proposing the alignment process is origin of the operation cell-voltage, and so, originates the voltage-range and voltage-profile. Considering the reacted-unreacted joint structures as a semiconductor junction^24–26,30,31^, the difference between Fermi energies of reacted and unreacted structures equals the external voltage of the cell^48^. Accordingly, while the Fermi levels move toward each other (during the Fermi alignment process), difference between the Fermi energies determines the external voltage. However, the electrochemical reaction would be initiated where the trans-band of lithiated structure starts to overlap with delithiated one. The reaction would be accomplished by full-overlap of the trans-bands. This process results in voltage-range, and then, voltage-profile. Therefore, as a matter of fact, width of trans-band (per eV) is as equal as the voltage-range (per V). Correspondence of the reacting electrons with the intercalating ions dictates that the voltage-profile corresponds to the outline of the trans-band. The starting (ending) voltage of the profile corresponds to the initiating (accomplishing) overlap of the intercalated and deintercalated trans-bands.

For simplicity, again, let us explain the concept with the case studies.

**Figure 2:**
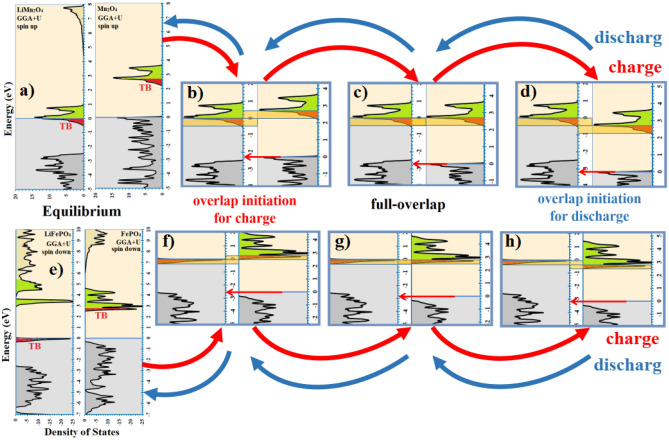
Schematic of Fermi levels’ alignment procedure of reacted-unreacted phases during charge and discharge process of LMO (**a**–**d**) and LFP (**e**–**h**) cathode materials, which causes voltage -range and -profile. The trans-bands (TBs) are highlighted by red. Difference between the Fermi energies equals to the external cell-voltage, which is indicated by the red arrow in each panel.

Figure 2 illustrates alignment process of the Fermi levels of intercalated and deintercalated LMO (Fig. 2a–d) and LFP (Fig. 2e–h). In the figure, the external voltage should be equal to difference between the Fermi energies. The charging reaction initiates when the overlap starts (Fig. 2b,f), and it will accomplish by the full overlap (Fig. 2c,g). Initiation and accomplishment of the discharging reactions are illustrated in Fig. 2d–h,c–g, respectively. Full DOS diagrams are given in S.I.

Equation (1) indicates that for each Li insertion, one electron should be transferred (also, a similar condition can be considered for LFP). Accordingly, when trans-band of the lithiated structure would be transferred above the Fermi level (unoccupied) completely, the reaction was accomplished. It corresponds to complete overlap of the trans-bands. Considering correlation between the energy and voltage, the width of the trans-band should be equal to the voltage-range for the evaluated electrodes. Consequently, the configure of the voltage-profile should be similar to the outline of the trans-band in the DOS diagram. In other words, transferring each electron between the deintercalated and intercalated trans-bands corresponds to (de)insertion of one Li^+^ ion. Therefore, outline of the trans-band should be coordinated with the voltage-capacity profile. Subsequently, maximum of the operational trans-band (peak of the band) relates to the maximum available capacity (it can be corresponded to the theoretical capacity, ${C}_{th}$).

**Figure 3:**
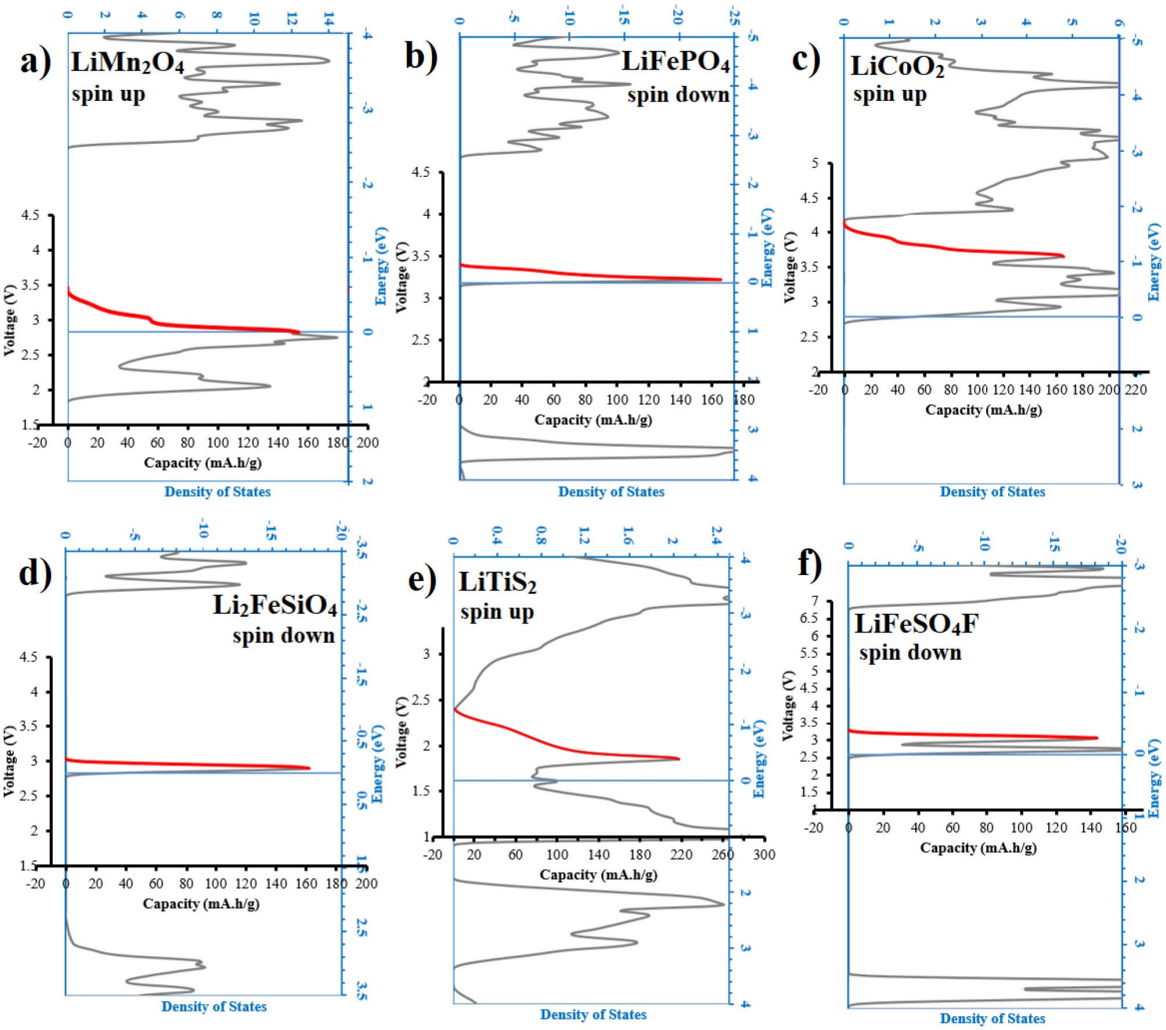
Calculated voltage-profiles and their accommodation with the operational trans-band in the DOS diagrams, proposed by the model, for different electrode materials, namely (**a**) LMO, (**b**) LFP, (**c**) LCO, (**d**) LFS, (**e**) TiS_2_, and (**f**) LFSF.

Figure 3 shows the voltage-capacity profiles for different electrode materials and their accommodation with the operational trans-band, as provided by the model.

As can be seen in Fig. 3a, configuration of the voltage-profile of LMO is in a fair agreement with the experimentally obtained profiles^49–51^. According to the reaction’s equation (Eq. 1), the valance state of Mn atoms (or half of them) just changes between + 3 and + 4. Therefore, the observed jag in the voltage plateau^49,50^ cannot be justified with different redox. Our approach precisely predicts configuration of the voltage plateau’s profile. The origin of the experimentally observed jag in the voltage plateau is due to corresponding jag in the DOS diagram of the trans-band (Figs. 1a and 3a).

The experimentally obtained voltage plateau for discharge reaction of LMO is 3.8–4.2 V ($\Delta V=0.4 V$)^52,53^. Noteworthy, Fig. 3 considers spontaneous reaction, i.e. discharge process, since the Fermi alignment should be energetically favorable. Calculated voltage -range and -profile via the model using different approximation methods was considered and performed. In our evaluations (see Table 1), we concluded that the Hybrid-Functional is the most accurate approximation for voltage values of LMO, since it predicted voltage-profile values of 3.5–3.9 V ($\Delta V=0.4 \text{V}$). This conclusion is in agreement with other studies^54,55^. Noteworthy, Fermi energy of metallic Li, i.e. 0.24 eV, should be taken into account^24^.

**Table 1:** Voltage-range, initial and final voltage values (V_up_ and V_down_) calculated by DFT methods as well as their related experimental values obtained from the cited references.

Material	Model					Experimental			
	Method	V_up_	V_down_	Voltage range	Voltage profile	References	V_up_	V_down_	Voltage range
LiMn_2_O_4_	GGA + U = 3 eV	3.3	2.9	0.4	Figure 3a	^49–51^	4.2	3.8	0.4
ZnMn_2_O_4_	GGA + U = 0.563 eV	2	1.6	0.4	Fig. S7	^81–85^	1.5	1.9	0.4
LiFePO_4_	GGA + U = 6 eV	3.45	3.15	0.3	Figure 3b	^6,7^	3.6	3.3	0.3
LiCoO_2_	GGA + U = 3.3 eV	4.1	3.7	0.4	Figure 3c	^10,56,57^	4.3	3.9	0.4
Li_2_FeSiO_4_	GGA + U = 4.5 eV	3	2.8	0.2	Figure 3d	^60–62^	2.93	2.74	0.19
LiTiS_2_	GGA + U = 5.5 eV	2.4	1.7	0.7	Figure 3e	^71–73^	2.4	1.8	0.6
LiFeSO_4_F	GGA + U = 8 eV	3.41	3.05	0.36	Figure 3f	^66–70^	3.70	3.33	0.37

The voltage-profile configurations are given in the cited figures.

Additionally, GGA + U (U = 3 eV) calculations predicted voltage-profile of 2.9–3.3 V ($\Delta V=0.4 \text{V}$) for LMO. However, LSDA + U (U = 3 eV) calculations showed more coincident with experimental voltage values (than GGA + U), i.e. 3.2–3.6 V ($\Delta V=0.4 \text{V}$). We also implemented different U values in DFT + U calculations to be sure for our final conclusions. The most accurate results ae reported in the paper. Noteworthy, in all of our evaluations, calculated voltage-range, i.e. width of trans-band, and voltage-profile configuration were almost the same for all the performed DFT + U calculations; however, some of the calculated initial and final voltage values did not coincide precisely with the experimental results.

Here, we conclude that to obtain the voltage-range and configuration of the voltage-profile, different approximation methods are reliable. In contrast, the initial and final voltage values were related to approximation methods. Our evaluations showed that GGA + U method was more reliable to predict voltage-profiles’ configuration.

Figure 3b shows the voltage-capacity profile of LFP. Considering width of the operational trans-band as the voltage-range, the approach predicts that the voltage-range of LFP should be equal to 0.3 V (Fig. 3b). According to Fig. 3b, taking into account also Fermi energy of metallic Li (0.24 eV), starting value of discharge voltage of LFP should be 3.45 V, and the ending value should be 3.15 V. These values are in a fair agreement with experimentally obtained values^6,7^.

Figure 3c illustrates voltage-profile of LCO. The observed small jag in the voltage-profile can be detected in experimentally obtained profiles^10,56,57^. Furthermore, initial and final voltage values of the plateau are in a good agreement with the experimentally^10,56,57^ obtained values (Table 1).

The theoretical capacity of LCO is 274 $\text{mAh }{\text{g}}^{-1}$, while the maximum available experimentally obtained capacity is 165 $\text{mAh }{\text{g}}^{-1}$. The peak value of the proposed operational trans-band within the DOS diagram of LCO (Fig. 3c) suggests that for the calculated voltage -range ($\Delta V=0.4 \text{V}$) and -profile, the maximum capacity should be 165 $\text{mAh }{\text{g}}^{-1}$. One may say that peak of the operational trans-band could determine practical available capacity. It has been established that more Li^+^ extraction could cause some distraction in the LCO structure^58,59^.

For orthorhombic polymorph of LFS (Li_2_FeSiO_4_), the calculated voltage-range was 0.2 V (2.8–3 V). It is in agreement with experimental measurements for this polymorph^60,61^. Noteworthy, existence of Li-Fe antisites increases width of the trans-band (see ref.^62^), which can cause to a wider voltage-range. Accordingly, in the case of observing a wider voltage-profile, it can be attributed to existence of the antisite defects. The antisite defects can produce two trans-bands in the lithiated DOS diagrams (see Fig. 3 of ref.^62^), which cause two-stage of voltage-profiles in the charging process. Interestingly, outline of trans-band of the delithiated structure suggests a continuous voltage-profile in discharge (see Fig. 5 of ref.^62^), which it is in a fair agreement with experimental observations. Notable, it seems that narrower voltage-range can be more favorable, since it ensures accomplishing the reaction before reaching cut-off voltage even in high rates^11,12^.

Interestingly, after extraction of two Li per formula another trans-band in spin up appears (see Figure S4c). This trans-band in the fully delithiated state (FeSiO_4_) is exactly above the Fermi level. It indicates that some reactions could take place in low voltage close to zero. It suggests that LFS can be also used as an anode. Surprisingly, we found a number of works which used LFS material as anode^63–65^. Evaluating those works, voltage-range of LFS as anode material was equal to related trans-band calculated for FeSiO_4_ (~ 0.5 V). These observations also validate the here proposed approach.

Moreover, the calculated voltage-profile plateau for the other polyanion cathode material, i.e. LFSF, is in a good agreement with experimental works^66–70^. A wider voltage-profile was obtained for TiS_2_ electrode material, which also its voltage values and plateau configuration coincide with the experimentals^71–73^.

Summary up, we checked the here proposed theoretical approach/model for a number of important case studies to confirm its validity. The results are reported in Table 1. The table indicates the reliability of the model. We stress again that the calculated voltage-range and configuration of the voltage-profile barely depend on the approximation method, while the calculation method can affect the initial and final voltage values of the profile.

As an additional point, this work, as a theoretical approach, considers the ideal situations^74–78^. To explain, it is worthy to note that the experimentally obtained voltage of a cell can be influenced by some practical parameters, i.e. overpotentials, influenced by kinetic factors, mostly C-rate (nominal current rate) or current density^11,12,22^. The practical voltage increases by enhancement of applied C-rate or actual (standardized/normalized) C-rate^27,43,79^. Accordingly, the considered experimental voltage values should be taken from the lowest available/reported C-rate data to be comparable with the calculated theoretical values. Notably, at the lowest C-rate, also, inactivation of the particles would be less probable^21,27,43,79^.

In addition, there are some other practical parameters which could slightly influence the final voltage (in mV), such as specific surface area, particle size, void, temperature, nature of conductive media, etc.^47^. However, from a theoretical point of view, these parameters should not be concerned; since, their effects would not be remarkable, and factually, are less than the calculations’ error^80^. Moreover, although they may cause a slight effect on the voltage value, the voltage -range and -profile configuration remain intact.

So far, voltage-profile has been calculated/estimated by combining thermodynamics with kinetics concepts using various approaches^19,45,86–88^. Subsequently, as a matter of fact, this paper is an attempt to extend the border of the modern thermodynamics toward kinetics’ territories; since, it implements pure concepts of modern physics to estimate the voltage-profile (a macroscopic measurable characterization). To determine situation of the approach in its related fields and to suggest a road-map for future studies, let’s illustrate further explanations and examples for the claimed potential benefits of the approach via a less formal language.

Obviously, the most straightforward benefits of the proposed approach are prediction and understanding the voltage -range and -profile of the well-known electrochemical systems, i.e. insertion batteries. It, per se, is a key benefit, since can help development of this state-of-art technology. However, it may not be end of the story. From a physicist point of view, the approach may provide some other side benefits.

Dealing solely with DOS diagrams (states of electrons) and ignoring consideration of ions and their kinetics, although trivial, once again, the approach evokes that the electron is the most important and considerable component in reaction between elements, in which its movement should be considered. Besides, here, this important abstract concept is visualized by correlating the electron states to the voltage-profile. Moreover, a step forward, this approach proposes that movement of states of the electron toward energy levels (i.e. trans-band in DOS) should also be considered (innovative insertion batteries provide it). Besides, interestingly, the approach is another attempt to convey macroscopic observations to the microscopic subjects and states. Consequently, it could be a solution of an important question, why is there a characteristic voltage-range for each component? In other words, why a reaction starts from a certain potential? Otherwise speaking, why are the electrochemical potentials quantized? Regardless of some existing (complicated) kinetic or thermodynamic methods, alternatively, the here approach offers a straightforward answer to the question. Simply, since energy of electrons is also quantized, i.e. quantum mechanics. Putting in another way, this study attempts to provide a methodology and tool to evaluate and understand measurable electrochemical characterizations by means of modern physics’ concepts/theories.

In addition, there is a tendency to introduce difference between Fermi energies (as a modern physics’ concept) as origin and motivation of the electrochemical potential (call it as *Fermi approach*)^24,46,47,89^, rather than classical thermodynamic concepts. In this way, this study suggests that the Fermi approach is a more powerful tool to justify experimental observations than its traditionally classical rivals, e.g. internal energy, and even, Gibbs free energy.

Furthermore, the proposed approach may bring to mind other conceptual subjects. While the DFT calculations perform for one unit-cell, they have been capable to predict behaviour of entire electrode body (even with many particles/grains), although it has been known^37,90–93^. Nevertheless, it has been known also that the intercalation procedure (or, generally, a reaction) would be performed in physically separated parts of the electrode body, i.e. via particle-by-particle or concurrent mechanisms etc.^11,21,29,86,94^. After all, the proposed approach suggests that (at least) all transition metals within the electrode body are in the same state at each SOC. Considering electrical connection between the particles, it evokes again mechanical quantum concepts. On this basis, all the transition metals in the electrode body would be in the same state of energy even if their neighbor atoms were different. This insight could be helpful for deep understanding of underlying mechanisms of the current insertion batteries, and even helping to design new generations of batteries.

Additionally, considering the cell-voltage related to energy difference between the reaction phases, this paper can be a clue to find origin of phase diagrams.

### Justification of different observed voltage profiles for charging and discharging

As it has been mentioned before, the external voltage equals to the difference between the Fermi energies of reacted and unreacted structures. Accordingly, increasing the voltage in the charge process corresponds to increasing distance between the Fermi energies. In Fig. 2b-d, assuming lithiated DOS diagram (LiMn_2_O_4_) to be motionless and delithiated one (Mn_2_O_4_) movable, the delithiated diagram should move downward. In this situation, the first overlapping should occur when the lowest trans-band of delithiated state meets the highest lithiated one. The controlling parameter should be the DOS bands with the lower states. Therefore, voltage-profile diagram of the charge process should be dictated by trans-band of the delithiated structure. On the other hand, in the discharge process, delithiated DOS diagram of Fig. 2b–d moves upward and its maximum operational trans-band meets the minimum state of the lithiated trans-band. Accordingly, operational trans-band of the lithiated structure should dictate discharge voltage-profile. Summery up, charging and discharging voltage-profile correspond to operational trans-band of the delithiated and lithiated structures, respectively.

In order to strength the concept, we checked voltage profile of Mn_2_O_4_ cathode material used in different intercalation cells. Interestingly, the charge voltage-profile barely depended on the intercalating ion, since it corresponds to operational trans-band of deintercalated structure, which it may just change slightly by the nature of the intercalating ion. The observed jag in the charge profile of LiMn_2_O_4_ can be seen also in various Mn_2_O_4_ metal-ion batteries (analogous with Fig. 1a, the green operational trans-band)^81–83,95–97^. Noteworthy, the crystal structure of MnO_2_ electrode has been various for different intercalation batteries; however, the same crystal structure (spinel) has been mostly used for Li and Zn -ion batteries. Accordingly, calculated charge/discharge voltage-profile of the ZnMn_2_O_4_, suggested by this approach, is given in the S.I, Figure S7, as an instance. The suggested profiles are in fair agreement with the experimental^81–85^ observations.

## Conclusions

This paper introduced new concepts, which regarded to the fields of electrochemistry, energy storage devices, and solid state physics (DFT). It was aimed to understand the underlying mechanisms of the intercalation batteries and analogous electrochemical systems.

To understand origin of the voltage behavior, the concepts of trans-band and operational trans-band were proposed. Alignment of the Fermi levels of the intercalated and deintercalated structures was the motivation of the reaction; whereas, during the alignment procedure, the difference between the Fermi levels equaled to the external cell-voltage. On this basis, while the Fermi levels of the intercalated-deintercalated structures was going to be aligned, the voltage-profile was started where the trans-band in the delithiated structure began to overlap with trans-band of lithiated structure. The final voltage corresponded to the whole overlap of the operational trans-bands. Accordingly, the voltage-range was attributed to the width of the operational trans-band. On the other hand, the outline of the deintercalated/intercalated operational trans-band determined configuration of the charge/discharge voltage-profile.

As an additional conclusion, the paper suggests that an appropriate electrochemical electrode material should have band gap, at least in one of its states (reacted/unreacted). This suggestion is analogous with the electrodes for solar cells. Accordingly, semiconductor cathode materials have advantage over conductive ones. However, there should be exceptions for anode materials which their cell-voltage was close to zero. In these cases, DOS value near the Fermi energy of reacted-unreacted structures determines available capacity at ${V}_{{Li/Li}^{+}}\approx 0$.

As a matter of fact, this paper was not a DFT study, but just used DFT calculations to establish higher concepts. Quantum mechanics have established that the electrons can just occupy specific levels of energy. In turn, DFT has established that the energy of electrons of many-body-systems (solid-state materials) is also quantized and show forbidden/allowed energy levels. Factually, this paper was an attempt to generalize the energy quantization concept and extend it to the reaction energies. Accordingly, it proposed that there could be forbidden/allowed energies for the reactions, at least, for insertion electrochemical reactions between the solids. This concept represented itself in the electrochemical point of view as voltage-range and voltage-profile. We indicated that the concept and its related calculations were successful to justify electrochemical behavior of the case studies. Furthermore, considering the cell-voltage, related to energy difference between the reaction phases, this paper can be a clue to find origin of phase diagrams.

Finally, this paper extended boundary of quantum mechanics toward territories of classical thermodynamics, and boundary of the modern thermodynamics toward kinetics.

### Supplementary Information

Supplementary Information.

## Data Availability

All data generated or analyzed during this study are included in this published article and its supplementary information files.

## References

[1] Liu G (2024). Controllable long-term lithium replenishment for enhancing energy density and cycle life of lithium-ion batteries. Energy Environ. Sci..

[2] Ye Y (2024). Quadruple the rate capability of high-energy batteries through a porous current collector design. Nat. Energy.

[3] Punyavathi R (2024). Sustainable power management in light electric vehicles with hybrid energy storage and machine learning control. Sci. Rep..

[4] Hu J-P (2020). High-rate layered cathode of lithium-ion batteries through regulating three-dimensional agglomerated structure. Energies.

[5] Zou L (2019). Lattice doping regulated interfacial reactions in cathode for enhanced cycling stability. Nat. Commun..

[6] Guan P (2020). Recent progress of surface coating on cathode materials for high-performance lithium-ion batteries. J. Energy Chem..

[7] Rowden B, Garcia-Araez N (2021). Estimating lithium-ion battery behavior from half-cell data. Energy Rep..

[8] Sasaki T, Ukyo Y, Novák P (2013). Memory effect in a lithium-ion battery. Nat. Mater..

[9] Cheng AL, Fuchs ER, Karplus VJ, Michalek JJ (2024). Electric vehicle battery chemistry affects supply chain disruption vulnerabilities. Nat. Commun..

[10] Li J, Zhao R, He X, Liu H (2009). Preparation of LiCoO 2 cathode materials from spent lithium–ion batteries. Ionics.

[11] Kalantarian MM, Yousefi Mashhour H (2020). Bipolarization of cathode particles as underlying mechanism for voltage hysteresis and the first charge cycle overvoltage of intercalation batteries. Electrochimica Acta.

[12] Haghipour A, TaherTalari M, Kalantarian MM (2022). Boundaries of charge-discharge curves of batteries. Sustain. Energy Fuels.

[13] Brunetti G (2011). Confirmation of the domino-cascade model by LiFePO_4_/FePO_4_ precession electron diffraction. Chem. Mater..

[14] Delmas C, Maccario M, Croguennec L, Le Cras F, Weill F (2008). Lithium deintercalation in LiFePO_4_ nanoparticles via a domino-cascade model. Nat. Mater..

[15] Padhi AK, Nanjundaswamy K, Goodenough JBD (1997). Phospho-olivines as positive-electrode materials for rechargeable lithium batteries. J. Electrochem. Soc..

[16] Srinivasan V, Newman J (2004). Discharge model for the lithium iron-phosphate electrode. J. Electrochem. Soc..

[17] Dargaville S, Farrell TW (2010). Predicting active material utilization in LiFePO_4_ electrodes using a multiscale mathematical model. J. Electrochem. Soc..

[18] Laffont L (2006). Study of the LiFePO_4_/FePO_4_ two-phase system by high-resolution electron energy loss spectroscopy. Chem. Mater..

[19] Malik R, Zhou F, Ceder G (2011). Kinetics of non-equilibrium lithium incorporation in LiFePO_4_. Nat. Mater..

[20] Yamada A (2006). Room-temperature miscibility gap in LixFePO_4_. Nat. Mater..

[21] Kalantarian MM (2014). Understanding non-ideal voltage behaviour of cathodes for lithium-ion batteries. J. Mater. Chem. A.

[22] Haghipour A, Momeni M, Yousefi-Mashhour H, Kalantarian MM (2022). Memory effects’ mechanism in the intercalation batteries: The particles’ bipolarization. ACS Appl. Mater. Interfaces..

[23] Safaeipour, S. & Kalantarian, M. M. Origin of Voltage Overshooting in Lithium-ion Batteries: nucleation influenced by particles’ bipolarization mechanism. *in press* (2024).

[24] Kalantarian MM, Haghipour A (2022). Estimation of electrochemical cell potentials and reaction energies using Fermi energies. Phys. Chem. Chem. Phys..

[25] Yousefi Mashhour H, Kalantarian MM (2021). A theoretical approach to evaluate and understand the electrical properties of the electrode materials of batteries. Phys. Chem. Chem. Phys..

[26] Kalantarian MM, Asgari S, Mustarelli P (2014). A theoretical approach to evaluate the rate capability of Li-ion battery cathode materials. J. Mater. Chem. A.

[27] Kalantarian MM, Yousefi-Mashhour H, Tahertalari M, Mustarelli P (2021). Standardization and normalization of capacity vs. current rate behavior of intercalation electrodes for Li-ion and Na-ion batteries. J. Energy Storage.

[28] Shahpouri E, Abavi-Torghabeh N, Kalantarian MM, Mustarelli P (2024). Normalization of charge/discharge time versus current rate diagrams for the rechargeable batteries. Phys. Chem. Chem. Phys..

[29] Safaeipour S, Shahpouri E, Kalantarian MM, Mustarelli P (2024). Inherent behavior of electrode materials of lithium‐ion batteries. ChemPlusChem.

[30] Momeni M, Mashhour HY, Kalantarian MM (2019). New approaches to consider electrical properties, band gaps and rate capability of same-structured cathode materials using density of states diagrams: Layered oxides as a case study. J. Alloys Compd..

[31] Kalantarian MM, Mashhour HY, Barjini MH (2020). A semi-quantitative approach to evaluate electrical rate-capability and conductivity of polyanion cathode materials of intercalation batteries using DOS diagrams. Ceram. Int..

[32] Blaha P, Schwarz K, Madsen G, Kvasnicka D, Luitz J (2001). WIEN2k, An Augmented Plane Wave Plus Local Orbitals Program for Calculating Crystal Properties.

[33] Hohenberg P, Kohn W (1964). Inhomogeneous electron gas. Phys. Rev..

[34] Björk H, Gustafsson T, Thomas JO, Lidin S, Petříček V (2003). Long-range ordering during delithiation of LiMn_2_O_4_ cathode material. J. Mater. Chem..

[35] Song B (2018). Metastable Li1+ δMn_2_O_4_ (0≤ δ≤ 1) spinel phases revealed by in operando neutron diffraction and first-principles calculations. Chem. Mater..

[36] Akimoto J, Gotoh Y, Oosawa Y (1998). Synthesis and structure refinement of LiCoO_2_ single crystals. J. Solid State Chem..

[37] Kalantarian MM, Asgari S, Mustarelli P (2013). Theoretical investigation of Li_2_MnSiO_4_ as a cathode material for Li-ion batteries: A DFT study. J. Mater. Chem. A.

[38] Tripathi R, Popov G, Sun X, Ryan DH, Nazar LF (2013). Ultra-rapid microwave synthesis of triplite LiFeSO_4_F. J. Mater. Chem. A.

[39] Zhou D (2018). Evolution of crystal structures and electronic properties for TiS_2_ at high pressure. J. Alloys Compd..

[40] Anisimov V, Solovyev I, Korotin M, Czyżyk M, Sawatzky G (1993). Density-functional theory and NiO photoemission spectra. Phys. Rev. B.

[41] Tran F, Blaha P, Schwarz K, Novák P (2006). Hybrid exchange-correlation energy functionals for strongly correlated electrons: Applications to transition-metal monoxides. Phys. Rev. B.

[42] Kalantarian MM, Hafizi Barjini M, Momeni M (2020). Ab initio study of AMBO_3_ (A = Li, Na and M = Mn, Fe Co, Ni) as cathode materials for Li-Ion and Na-Ion batteries. ACS Omega.

[43] Kalantarian MM, Yousefi Mashhour H, Shahroudi H, Osanloo N, Mustarelli P (2020). Insight into charge/discharge behaviours of intercalation cathode materials: relation of delivered capacity versus applied rate and analysis of multi-particle intercalation mechanisms. Phys. Chem. Chem. Phys..

[44] Kalantarian MM, TaherTalari M, Yousefi-Mashhour H (2021). An insight into charge/discharge behaviors of lithium/sodium-sulfur conversion batteries and their similarity to the lithium/sodium-ion intercalation batteries: Relation of delivered capacity vs current rate. Energy Storage.

[45] Gao J, Shi S-Q, Li H (2015). Brief overview of electrochemical potential in lithium ion batteries. Chin. Phys. B.

[46] Goodenough JB, Kim Y (2011). Challenges for rechargeable batteries. J. Power Sour..

[47] Safaeipour, S. & Kalantarian, M. M. Origin of the electrochemical potential: Fermi vs. Gibbs Free Energies. *Nano-Structures & Nano-Objects ***39**, 101213. 10.1016/j.nanoso.2024.101213 (2024).

[48] RayChaudhuri B (2011). On the determination of the emission wavelength of an infrared LED with common laboratory instruments. Eur. J. Phys..

[49] Yu F (2022). Spinel LiMn2O4 cathode materials in wide voltage window: Single-crystalline versus polycrystalline. Crystals.

[50] Tang D (2014). Electrochemical behavior and surface structural change of LiMn_2_O_4_ charged to 5.1 V. J. Mater. Chem. A.

[51] Li S, Lei D, Xue Y, Geng S, Cui X (2017). One-step solid-state synthesis of nanosized LiMn_2_O_4_ cathode material with power properties. Ionics.

[52] Kim S-W (2011). Preparation and electrochemical characterization of doped spinel LiMn 1.88 Ge 0.1 Li 0.02 O_4_ cathode material. Electron. Mater. Lett..

[53] Amos CD, Roldan MA, Varela M, Goodenough JB, Ferreira PJ (2016). Revealing the reconstructed surface of Li [Mn_2_]O_4_. Nano Lett..

[54] Eckhoff M, Blöchl PE, Behler J (2020). Hybrid density functional theory benchmark study on lithium manganese oxides. Phys. Rev. B.

[55] Young MJ, Schnabel HD, Holder AM, George SM, Musgrave CB (2016). Band diagram and rate analysis of thin film spinel LiMn_2_O_4_ formed by electrochemical conversion of ALD-grown MnO. Adv. Funct. Mater..

[56] Liu X (2020). Conformal prelithiation nanoshell on LiCoO_2_ enabling high-energy lithium-ion batteries. Nano Lett..

[57] Wang Z (2014). Structure and electrochemical performance of LiCoO_2_ cathode material in different voltage ranges. Ionics.

[58] Dokko K (2000). In situ observation of LiNiO_2_ single-particle fracture during Li-Ion extraction and insertion. Electrochem. Solid-State Lett..

[59] Liu Q (2018). Approaching the capacity limit of lithium cobalt oxide in lithium ion batteries via lanthanum and aluminium doping. Nat. Energy.

[60] Nytén A, Abouimrane A, Armand M, Gustafsson T, Thomas JO (2005). Electrochemical performance of Li_2_ FeSiO_4_ as a new Li-battery cathode material. Electrochem. Commun..

[61] Nytén A, Kamali S, Häggström L, Gustafsson T, Thomas JO (2006). The lithium extraction/insertion mechanism in Li_2_FeSiO_4_. J. Mater. Chem..

[62] Lu X (2016). Density functional theory insights into the structural stability and Li diffusion properties of monoclinic and orthorhombic Li_2_FeSiO_4_ cathodes. J. Power Sour..

[63] Liang E-Q (2017). Electrochemical performance of Li_2_FeSiO_4_ as anode material for lithium-ion batteries. Int. J. Electrochem. Sci..

[64] Hsu C-H, Du T-R, Tsao C-H, Lin H-P, Kuo P-L (2019). Hollow Li_2_FeSiO_4_ spheres as cathode and anode material for lithium-ion battery. J. Alloys Compd..

[65] Xu Y, Li Y, Liu S, Li H, Liu Y (2012). Nanoparticle Li_2_FeSiO_4_ as anode material for lithium-ion batteries. J. Power Sour..

[66] Tripathi R, Ramesh T, Ellis BL, Nazar LF (2010). Scalable synthesis of tavorite LiFeSO_4_F and NaFeSO_4_F cathode materials. Angewandte Chemie.

[67] Sobkowiak A (2013). Understanding and controlling the surface chemistry of LiFeSO_4_F for an enhanced cathode functionality. Chem. Mater..

[68] Blidberg A (2017). Identifying the electrochemical processes in LiFeSO_4_F cathodes for lithium ion batteries. ChemElectroChem.

[69] Kim M, Jung Y, Kang B (2015). High electrochemical performance of 3.9 V LiFeSO_4_F directly synthesized by a scalable solid-state reaction within 1 h. J. Mater. Chem. A.

[70] Recham N (2009). A 3.6 V lithium-based fluorosulphate insertion positive electrode for lithium-ion batteries. Nat. Mater..

[71] Zhang L (2018). Tracking the chemical and structural evolution of the TiS_2_ electrode in the lithium-ion cell using operando X-ray absorption spectroscopy. Nano Lett..

[72] Dehghan P (2023). Growth of TiS_2_ nanoflakes using CVD approach for sodium-ion battery application. J. Nanoparticle Res..

[73] Chaturvedi A, Hu P, Aravindan V, Kloc C, Madhavi S (2017). Unveiling two-dimensional TiS_2_ as an insertion host for the construction of high energy Li-ion capacitors. J. Mater. Chem. A.

[74] Siriwardane EM, Demiroglu I, Sevik C, Peeters FM, Çakır D (2020). Assessment of sulfur-functionalized mxenes for li-ion battery applications. J. Phys. Chem. C.

[75] Benzidi H (2019). Arsenene monolayer as an outstanding anode material for (Li/Na/Mg)-ion batteries: Density functional theory. Phys. Chem. Chem. Phys..

[76] Urban A, Seo D-H, Ceder G (2016). Computational understanding of Li-ion batteries. npj Comput. Mater..

[77] Longo R (2014). Phase stability of Li–Mn–O oxides as cathode materials for Li-ion batteries: Insights from ab initio calculations. Phys. Chem. Chem. Phys..

[78] Alfaruqi MH (2020). Density functional theory investigation of mixed transition metals in olivine and tavorite cathode materials for Li-ion batteries. ACS Appl. Mater. Interfaces.

[79] Shahpouri E, Abavi-Torghabeh N, Kalantarian MM, Mustarelli P (2024). Normalization of charge/discharge time versus current rate diagrams for rechargeable batteries. Phys. Chem. Chem. Phys..

[80] Mertin GK (2021). On the possible influence of the Fermi-Dirac statistics on the potential and entropy of galvanic cells. J. Power Sour..

[81] Sun W (2017). Zn/MnO_2_ battery chemistry with H^+^ and Zn^2+^ coinsertion. J. Am. Chem. Soc..

[82] Yuan Y (2022). Understanding intercalation chemistry for sustainable aqueous zinc–manganese dioxide batteries. Nat. Sustain..

[83] Zhang N (2017). Rechargeable aqueous zinc-manganese dioxide batteries with high energy and power densities. Nat. Commun..

[84] Alfaruqi MH (2018). Structural transformation and electrochemical study of layered MnO_2_ in rechargeable aqueous zinc-ion battery. Electrochimica Acta.

[85] Duan Q, Wang Y, Dong S, Denis Y (2022). Facile electrode additive stabilizes structure of electrolytic MnO_2_ for mild aqueous rechargeable zinc-ion battery. J. Power Sour..

[86] Dreyer W (2010). The thermodynamic origin of hysteresis in insertion batteries. Nat. Mater..

[87] Roscher MA, Bohlen O, Vetter J (2011). OCV hysteresis in Li-ion batteries including two-phase transition materials. Int. J. Electrochem..

[88] Lin Y, Zheng J, Wang C, Qi Y (2020). The origin of the two-plateaued or one-plateaued open circuit voltage in Li–S batteries. Nano Energy.

[89] Goodenough JB, Kim Y (2010). Challenges for rechargeable Li batteries. Chem. Mater..

[90] Shahpouri E (2024). Evaluation of Ti_3_C_2_ as electrode material for Li, Na, Mg, Al, K, Ca, and Zn-ion intercalation batteries: A DFT study. Results Chem..

[91] Yousefi-Mashhour, H. *et al.* Unlocking the power of lithium trifluoride, LiMF3 (M = Mn Co, Fe, Ni, and V), materials through DFT: A paradigm shift in electrode candidates for high-performance Li-ion batteries. *Ionics,* 1–14. 10.1007/s11581-024-05506-4 (2024).

[92] Yousefi-Mashhour H, Safaeipour S, Hassani S, Kalantarian MM, Namiranian A (2024). Exploring the potential of lithium metal oxyfluoride, LiMOF, compounds (M = Mn, Fe, Co, and Ni) for advanced li-ion battery applications: A comprehensive Ab initio investigation. J. Phys. Chem. C.

[93] Safaeipour S, Kalantarian MM, Shabani MO, Faeghinia A (2024). Investigation on TiS_2_ electrode material for intercalation batteries, namely Li, Na, Mg, Al, K, Ca, and Zn-ion cells: A DFT study. Results Chem..

[94] Li Y (2014). Current-induced transition from particle-by-particle to concurrent intercalation in phase-separating battery electrodes. Nat. Mater..

[95] Yuan C (2014). Investigation of the intercalation of polyvalent cations (Mg^2+^, Zn^2+^) into λ-MnO_2_ for rechargeable aqueous battery. Electrochimica Acta.

[96] Wang Z (2021). Reduced graphene oxide thin layer induced lattice distortion in high crystalline MnO_2_ nanowires for high-performance sodium-and potassium-ion batteries and capacitors. Carbon.

[97] Nam KW (2015). The high performance of crystal water containing manganese birnessite cathodes for magnesium batteries. Nano Lett..

