# Supplementary material for: Origin of electrochemical voltage range and voltage profile of insertion electrodes
Source: Sci Rep. 2024 Jun 21;14:14311. doi: 10.1038/s41598-024-65230-x (PMC11192894; doi:10.1038/s41598-024-65230-x)
Supplement: Supplementary file 1 — Supplementary Information. [file 41598_2024_65230_MOESM1_ESM.pdf]

Supporting Information

Origin of electrochemical voltage range and voltage profile of insertion electrodes

Elham Shahpouri[a], Mohammad Mahdi Kalantarian[a]*

[a] Department of Ceramic, Materials and Energy Research Center, PO Box 31787-316, Karaj, Iran.
E-mails: kalantarian@gmail.com, m.kalantarian@merc.ac.ir

Supporting information for this article is given via a link at the end of the document.

Keywords: Operating potential range; voltage window; intercalation batteries' electrode; underlying mechanism; voltage profile

S1. Methodology details

In the WIEN2K code, for each atom, a sphere radius around it should be considered, called the muffin-tin radius, i.e. RMT. Inside the non-overlapping spheres of RMT around each atom, linear combinations of radial solutions of the Schrödinger equation times the spherical harmonics were used and the plane-wave basis set was used in the interstitial region. Spheres of the RMT around each atom were dictated by the structure of each material. The RMT values are given in Table S1. The electron and spin configuration of the atoms were software defaults and were: Li: [He] $2s^1$, O: [He] $2s^2$ $2p^4$, F: [He] $2s^2$ $2p^5$, Si: [Ne] $3s^2$ $3p^2$, P: [Ne] $3s^2$ $3p^3$, S: [Ne] $3s^2$ $3p^4$, Fe_{up}: [Ar] $3d^{6.5}$(4.5m, 2 k) $4s^{1.5}$(1m, 0.5 k), Mn_{up}: [Ar] $3d^5$(3m, 2 k) $4s^2$(1m, 1k), Ti_{up}: [Ar] $3d^2$(2m, 0k) $4s^2$(1m, 1k), and Co_{up}: [Ar] $3d^7$ (5m, 2 k) $4s^2$ (1m, 1 k).

Table S1. Used RMT values (a.u) for different evaluated electrode materials.

Material	Li	O	F	Si	P	S	Ti	Mn	Fe	Co	Zn
$LiMn_2O_4$	1.76	1.42	-	-	-	-	-	2	-	-	-
$ZnMn_2O_4$	-	1.42	-	-	-	-	-	2	-	-	2
$LiFePO_4$	1.97	1.42	-	-	1.42	-	-	-	2	-	-
$LiCoO_2$	1.75	1.42	-	-	-	-	-	-	-	2.02	-
Li_2FeSiO_4	1.6	1.45	-	1.45	-	-	-	-	1.72	-	-
$LiTiS_2$	2.07	-	-	-	-	2	2.32	-	-	-	-
$LiFeSO_4F$	1.82	1.35	1.74	-	-	1.35	-	-	1.96	-	-

S2. Supplementary figures

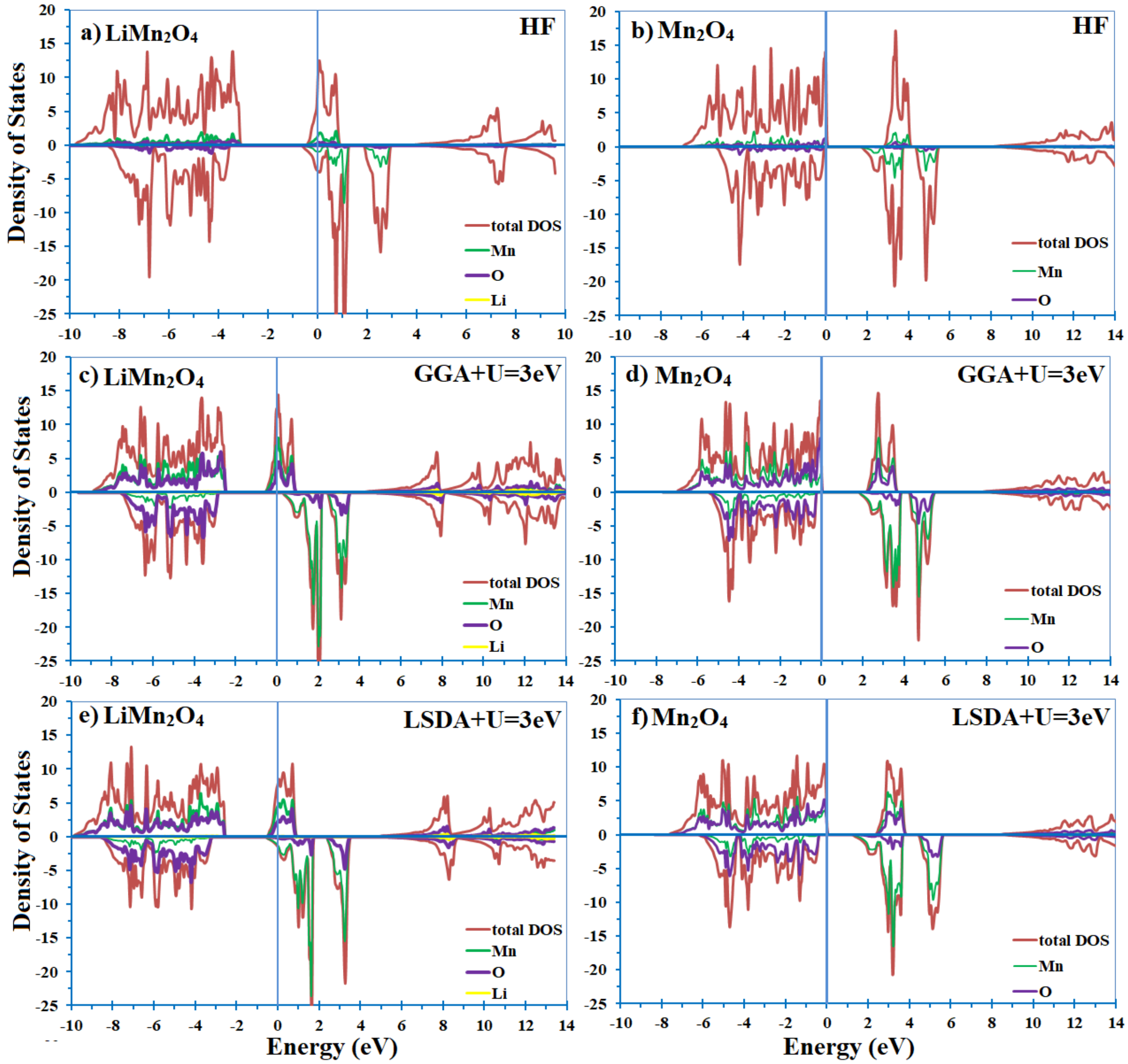

Figure S1. Full DOS diagrams of $LiMn_2O_4$ (lithiated) and Mn_2O_4 (delithiated), calculated by Hybrid functional (HF, panels a and b), GGA+U=3eV (panels c and d), and LSDA+U=3eV (panels e and f) DFT methods.

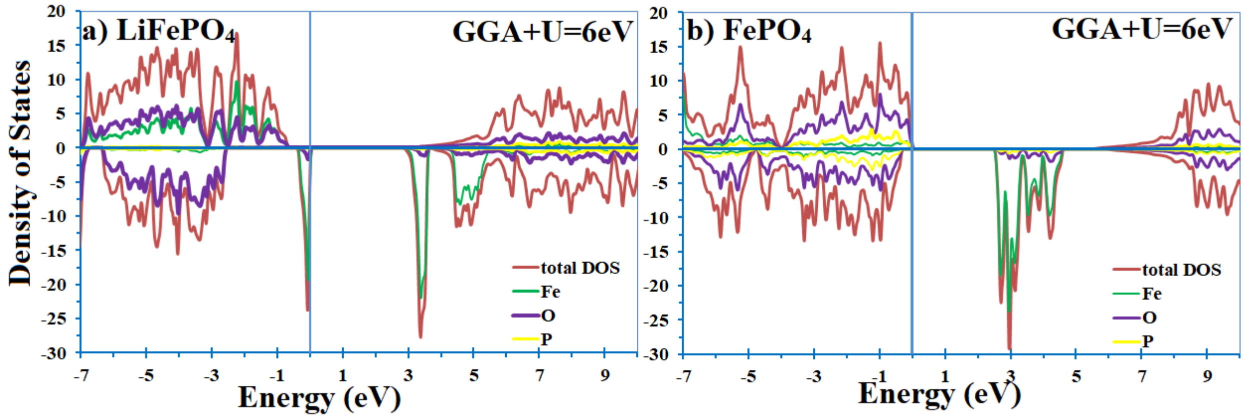

Figure S2. Full DOS diagrams of $LiFePO_4$ (lithiated, panel a) and $FePO_4$ (delithiated, panel b), calculated by GGA+U=6eV DFT method.

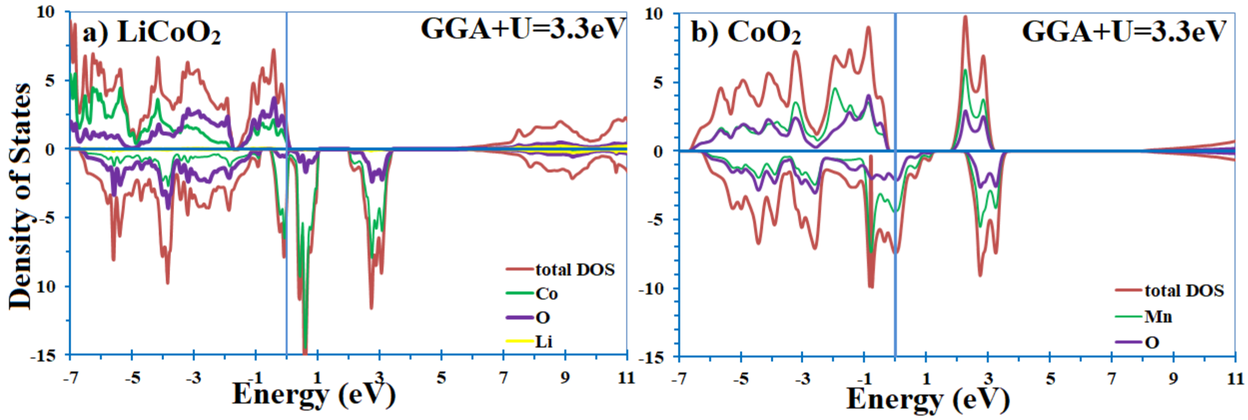

Figure S3. Full DOS diagrams of $LiCoO_2$ (lithiated, panel a) and CoO_2 (delithiated, panel b), calculated by GGA+U=3.3eV DFT method.

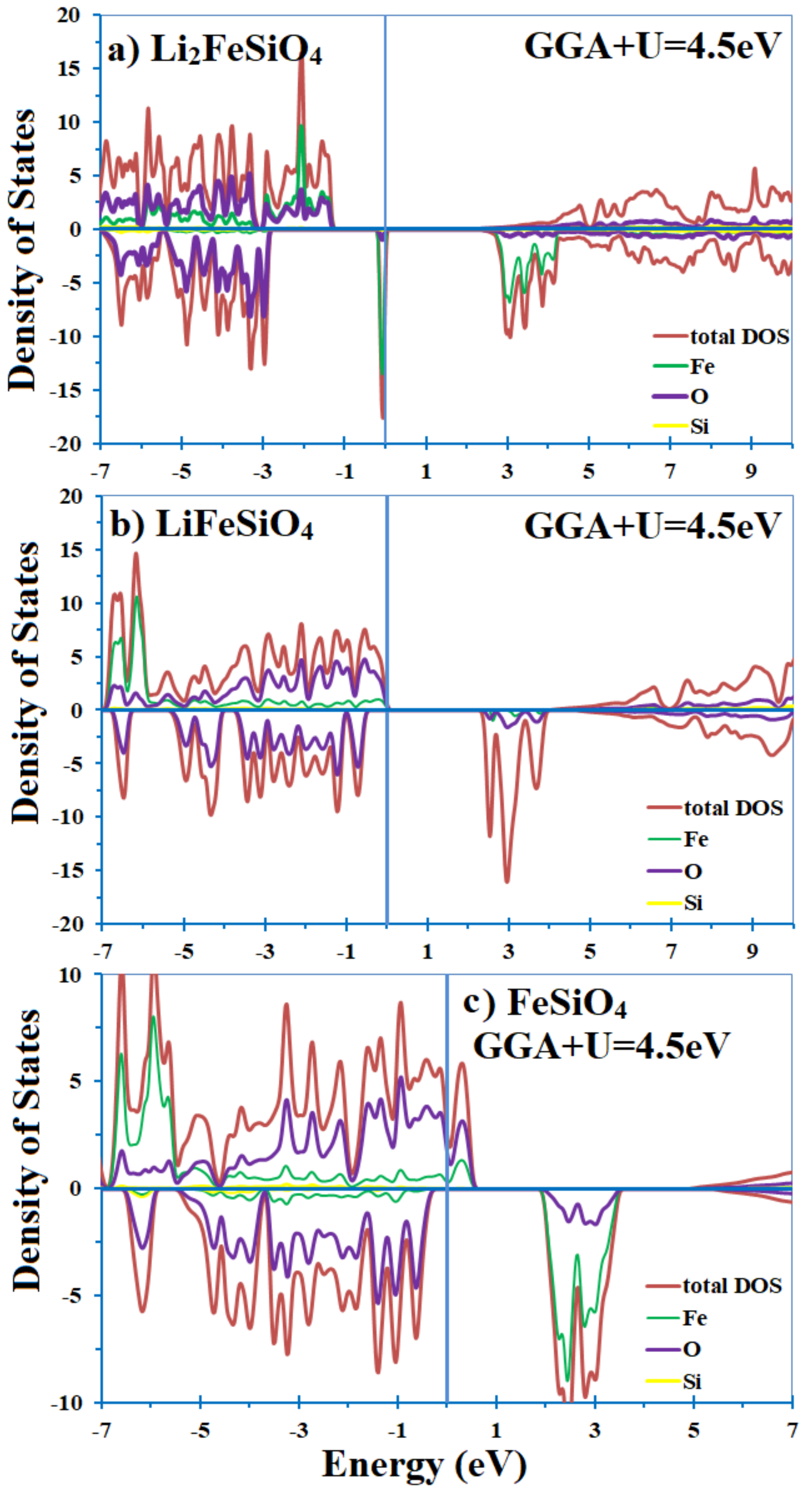

Figure S4. Full DOS diagrams of Li_2FeSiO_4 (panel a), $LiFeSiO_4$ (panel b), and $FeSiO_4$ (panel c) calculated by GGA+U=4.5eV DFT method.

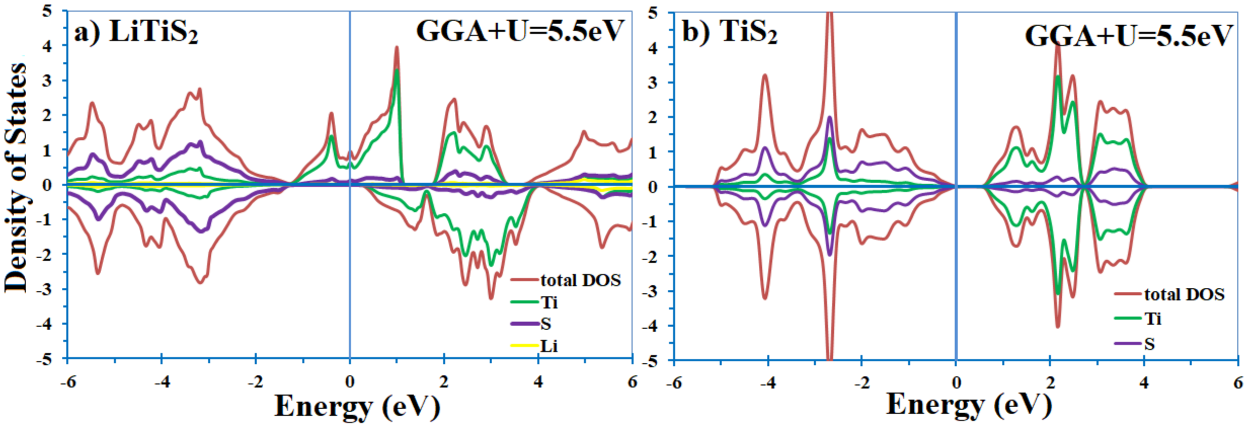

Figure S5. Full DOS diagrams of $LiTiS_2$ (lithiated, panel a) and TiS_2 (delithiated, panel b), calculated by GGA+U=5.5eV DFT method.

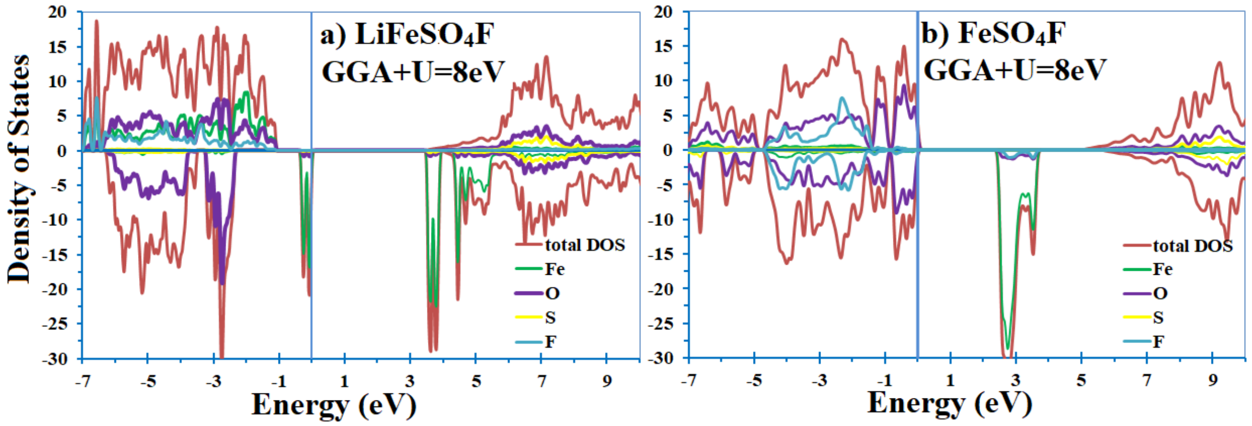

Figure S6. Full DOS diagrams of $LiFeSO_4F$ (lithiated, panel a) and $FeSiO_4F$ (delithiated, panel b), calculated by GGA+U=8eV DFT method.

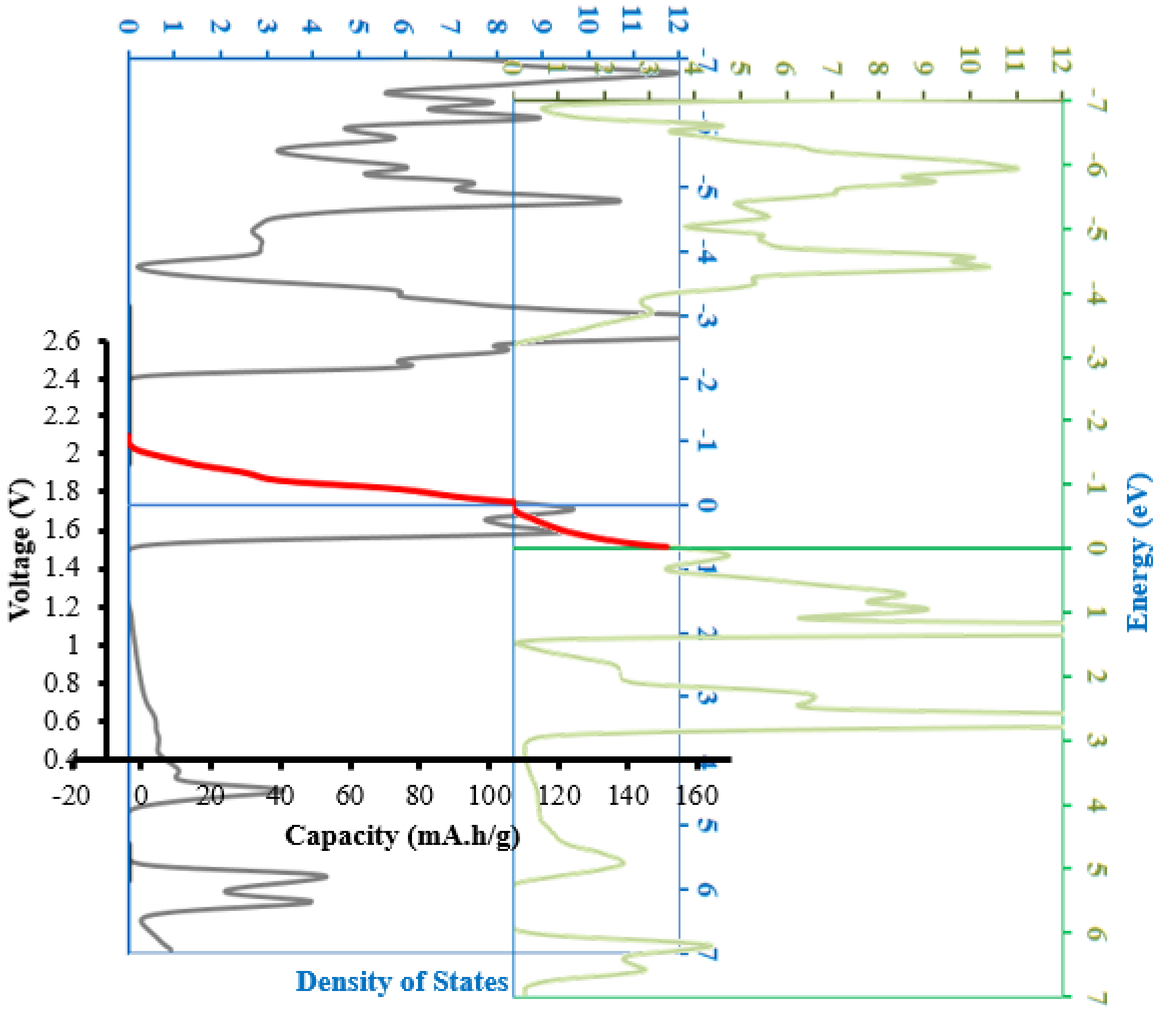

Figure S7. calculated voltage profile and its accommodation with the operational trans-band in the DOS diagrams proposed by the model for $ZnMn_2O_4$
